# The effect of autistic traits on disembedding and mental rotation in neurotypical women and men

**DOI:** 10.1038/s41598-022-08497-2

**Published:** 2022-03-17

**Authors:** Massimiliano Conson, Vincenzo Paolo Senese, Isa Zappullo, Chiara Baiano, Varun Warrier, The LabNPEE Group, Angelo Barone, Roberta Cecere, Andrea Cisone, Roberta Cerrone, Ylenia Crocetto, Lea Dell’Aversana, Alessia Delle Curti, Alessandro Fontana, Concetta Fusotto, Giusi Mautone, Generosa Montuori, Monica Positano, Gennaro Raimo, Annamaria Raiola, Maria Russo, Federica Sacco, Maria Sarno, Angela Sepe, Alessandro Troise, Simona Raimo, Barbara Rauso, Sara Salzano, Simon Baron-Cohen

**Affiliations:** 1 Department of Psychology, University of Campania Luigi Vanvitelli, Caserta, Italy; 2 Studies of Integrated Neuropsychological Therapy, Salerno, Italy; 3 Autism Research Centre, Department of Psychiatry, University of Cambridge, Douglas House, 18B Trumpington Road, Cambridge, CB2 8AH UK; 4 Cognitive-Behavioral School of Psychotherapy “Serapide SPEE”, Naples, Italy

**Keywords:** Psychology, Human behaviour

## Abstract

Recent data has revealed dissociations between social and non-social skills in both autistic and neurotypical populations. In the present study, we investigated whether specific visuospatial abilities, such as figure disembedding and mental rotation, are differently related to social and non-social autistic traits, in neurotypical women and men. University students (N = 426) completed the Autism Spectrum Quotient (AQ), figure disembedding and mental rotation of two-dimensional figures tasks. AQ social skills (AQ-social) and attention-to-details (AQ-attention) subscales were used as measures of social and non-social autistic traits, respectively. Mental rotation was affected by a significant interaction between sex, social and non-social traits. When non-social traits were above the mean (+ 1 SD), no sex differences in mental rotation were found. Instead, below this value, sex differences depended on the social traits, with men on average outperforming women at middle-to-high social traits, and with a comparable performance, and with women on average outperforming men, at lower social traits. A small positive correlation between figure disembedding and social traits was observed in the overall sample. These results are interpreted in terms of the hyper-systemizing theory of autism and contribute to the evidence of individual differences in the cognitive style of autistic people and neurotypical people with autistic traits.

## Introduction

The issue of whether visuospatial abilities are superior in autistic people compared to neurotypical people has been debated for several decades^1–3^ and continues to attract the attention of researchers^4,5^. Among the visuospatial tasks most expected to reveal differences between autistic and neurotypical people are figure disembedding and mental rotation, although there are conflicting results due to methodological differences across the studies^6^. Such discrepancies also appear when investigating the cognitive profile of neurotypical individuals in relation to their number of autistic traits.

Autistic traits exist across the general population, with autism at the extreme end of a quantitative distribution^7,8^. Neurotypical adults with high levels of autistic traits on average show superior performance compared to people with low levels of autistic traits on figure disembedding tasks, analogous to what has been found in autistic people^9–11^. Larger inconsistencies are seen on mental rotation^12–15^. Some studies have found that different cognitive abilities and autistic traits predict mental rotation performance in women and men^14^. The interaction between autistic traits and sex could lead to differences both in performance^12^ and the strategy used to solve mental rotation tasks^15^. Other studies have found that an interaction between autistic traits and the academic degree subject could account for differences in mental rotation in men and women^13^.

Together, these results converge in highlighting the need to test the relationship between autistic traits and mental rotation taking into account sex differences. Sex differences in mental rotation have been investigated for a long time, with some authors reporting better abilities on average in men than women, although the size of the difference varies across studies^16–20^, while other authors reporting no difference at all^13,21,22^. Analogously, some discrepancies in the literature on sex differences in figure disembedding are present but are partially accounted for by age. Indeed, in children, significant sex differences do not emerge so clearly^23,24^ or a non-significant advantage of boys over girls has been observed^25,26^, while in adults the lack of significant sex differences seems more consistent^27–29^.

Growing data are revealing dissociations between social and non-social domains in autism and neurotypical people^8,30–33^, strengthening the view that a continuity exists between autism and autistic traits in the general population^7,34,35^. In a study exploring whether social or non-social autistic traits relate to figure disembedding ability, Russell-Smith et al.^11^ used the Autism Spectrum Quotient (AQ^7^) and applied a two-factor model of the measure to differentiate social traits (“social difficulties”) and non-social traits (“details/patterns”)^36^. Results showed that superior performance in figure disembedding was related to greater social traits. Consistently, DiCriscio and Troiani^37^ showed that performance on a figure disembedding task positively correlated with autistic-like social features, as assessed by the Broader Autism Phenotype Questionnaire^38^, although this result was observed in men but not in women. Since figure disembedding implies ignoring the gestalt and focusing on local details, these findings provide support for the view that heightened focus on details, together with weak gestalt processing (weak central coherence), could prevent a person from integrating the social cues that construct meaning in a context^39^.

Until now, the distinction between social and non-social traits has not been considered in studies investigating the influence of autistic traits on mental rotation. It is worth noting that better mental rotation abilities have been related to high systemizing^12–14^. Systemizing, defined as the drive to analyse, understand and build systems, a strength in autistic individuals (hyper-systemizing^40^), is one of the non-social aspects of autism^31^. Mental rotation, requiring the person to deal with the visual stimulus as a variable that can be transformed (rotated) to predict how it will appear, has been considered a measure of systemizing^17,41^. Indeed, systemizing has been operationalised as *‘if-and-then’* logical reasoning^42^ and mental rotation nicely illustrates this: ‘*If* I take a vertical shape, *and* I rotate it 90 degrees anti-clockwise, *then* it will appear horizontal’.

Hence, available data allow us to envisage the possibility that figure disembedding and mental rotation abilities are differently related to social and non-social autistic traits, with figure disembedding most relating to social traits and mental rotation to non-social traits, and with these relationships being moderated by sex. In the present study, we aimed to test this hypothesis on a sample of Italian university students by assessing figure disembedding abilities and mental rotation through well-validated paper-and-pencil tasks^43,44^ and autistic traits as measured by the AQ^7,45^.

Different factor analyses of the AQ identified from two to five factors, almost all comprising a social-interaction factor and an attention-to-detail factor, although psychometric evaluations of the AQ have led to disagreement as to how these factors are defined^46^. Palmer et al.^47^ performed a cluster analysis on a large data set of general-population adults and identified two subgroups with different trait profiles: one group with greater social difficulties and lower detail orientation, and the other group with lesser social difficulties and greater detail orientation. Moreover, the authors extracted a three-factor structure from AQ, with sociability and mentalising factors sharing moderate-to-strong positive correlations with the AQ’s social skills, imagination, communication and attention switching subscales, but correlating negatively with the attention-to-detail subscale. In contrast, the detail orientation factor showed a strong positive correlation with the AQ’s attention-to-detail subscale and very weak correlations with social skill, attention switching, communication, and imagination subscales. Hence, Palmer et al.’s^47^ results suggest considering social and attention-to-detail trait domains independently, a perspective also shared by other authors^48,49^. More recently, English et al.^46^ performed confirmatory factor analyses across competing factor models of the AQ and results supported Russell-Smith et al.’s^36^ model differentiating three factors: social skill; details and patterns; communication and mindreading.

Due to the complexity in identifying the most psychometrically sound factor structure of the AQ, here we used Baron-Cohen et al.’s^7^ AQ social skills (AQ-social) and attention-to-details (AQ-attention) subscales as separate variables representing the two core clusters of respectively social and non-social autistic traits and with respect to which the validity has been verified for the Italian version of the scale^45^.

## Methods

### Participants

Participants were 426 Italian native speaker students (206 women, 220 men: mean age = 23.75 years, SD = 2.04) enrolled from different Universities in the Campania region (Southern Italy). All participants were recruited from systemizing-based degree subjects, as computer science, engineering, mathematics, chemistry, physics, biology, law, economics, languages and philosophy (for a detailed description of the degree subjects see Ref.^13^).

Individuals could participate in the study if they did not report any past or current history of neurological or psychiatric conditions. Individuals with a diagnosis of neurodevelopmental disorder or reporting past or current use of substances acting on the central nervous system were also excluded from the study.

The study was approved by the Ethic Committee of the Department of Psychology of the University of Campania Luigi Vanvitelli and was conducted in accordance with the ethical standards of Helsinki Declaration; written informed consent was obtained from all participants prior to testing.

Materials underpinning the research, and the datasets generated and analysed during the current study are available from the corresponding author (S.B.C.) on request.

### Measures

#### Autistic traits

Autistic traits were assessed via the Italian version of the Autism Spectrum Quotient (AQ^45^). The AQ measures, both in clinical and non-clinical samples, the individual’s autistic traits in five domains: social skill; attention switching; attention-to-detail; communication; imagination. All the participants completed the full 50-item AQ, and the results were scored according to Baron-Cohen et al.’s^7^ criteria. For the present study, total AQ score and five scores for the main subscales (higher scores always implies stronger autistic traits) were obtained and used to test sex differences. However, as underscored above, to pursue the main aim of the study, we used Baron-Cohen et al.’s^7^ AQ social skills (AQ-social) and attention-to-details (AQ-attention) subscales as variables representing social and non-social autistic traits, respectively.

#### Visuospatial abilities

**Figure 1:**
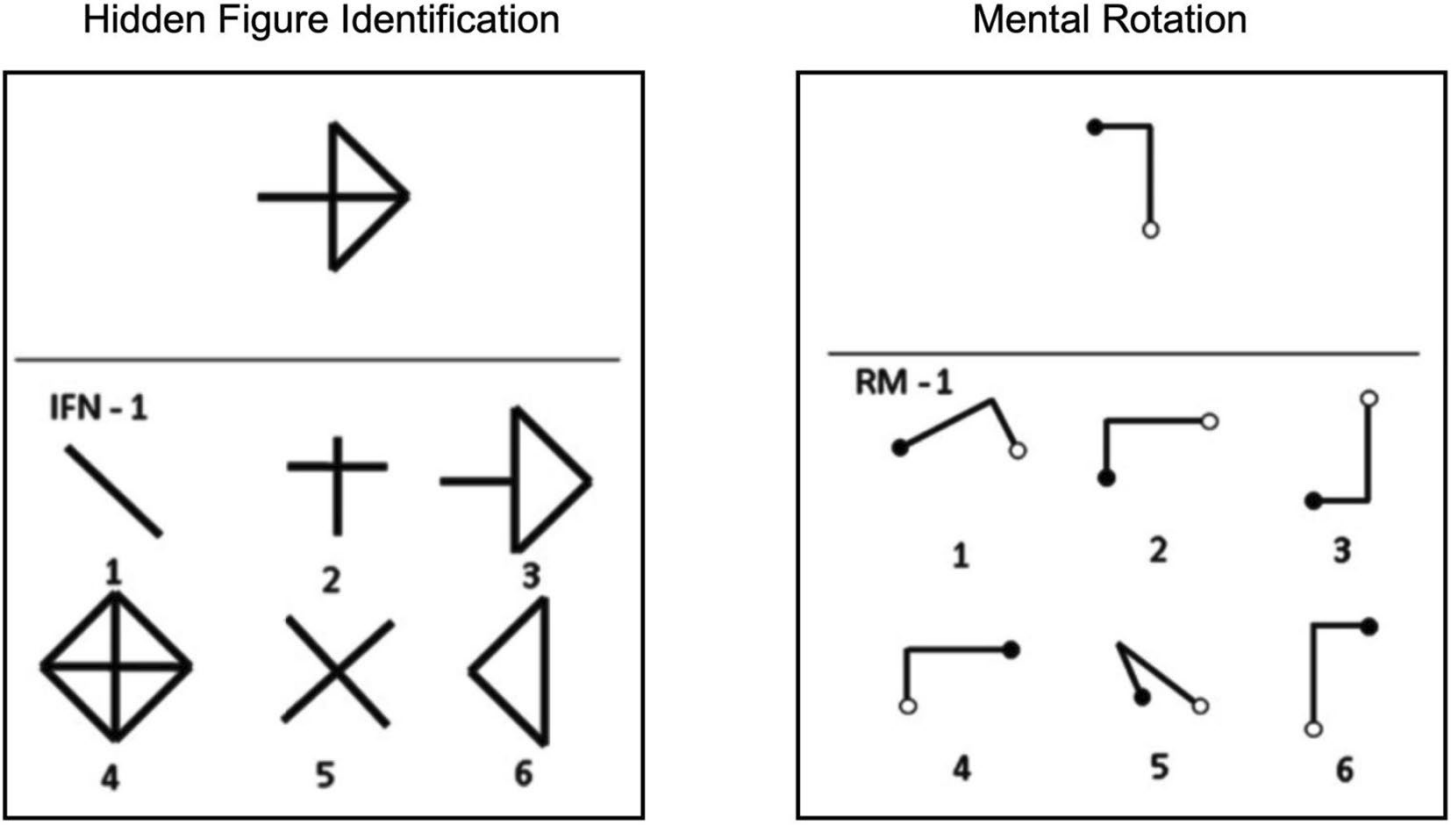
Examples of items from Hidden Figure Identification and Mental Rotation tasks. In the Hidden Figure Identification task, participants have to identify among the options a simple shape which is contained within the target stimulus (here, the correct response is the option 3). In the Mental Rotation task, participants are required to identify among the options the one matching the target stimulus after a mental rotation on the horizontal plane (here, the correct response is the option 2).

Figure disembedding and mental rotation were evaluated by two paper-and-pencil tests derived from a well-validated battery developed for assessing visuospatial abilities^43,44^. Both tasks consist of a series of items, each presented on a paper sheet. For each item, the target stimulus is placed in the upper part of the sheet while the options are placed within a six-choice display in the lower part of the sheet (Fig. 1). Items are presented one at a time and participants are required to indicate among the six options the only one corresponding to the stimulus target, without time constraints. To give their answer, the participants have to verbally report the number corresponding to the selected option. Two practice items are given before the task in order to be sure that the participant clearly understands the instructions.

Both correct responses and the total time needed to solve all the items are recorded. Scoring procedures assign one point for each correct response; no penalty is computed for wrong responses. Following previous studies^43,44^ showing that for both figure disembedding and mental rotation tasks the accuracy parameter provides a reliable (person separation indices > 0.81) and valid measure of the two visuospatial abilities, here statistical analysis took into account only response accuracy.

All participants were assessed individually in a dedicated room, away from sources of noise or other distractions.

##### Figure disembedding

In the Hidden Figure Identification task^43,44^ participants are presented with a target stimulus and six figures (i.e., the correct response and five distractors) consisting of complex geometrical shapes. The task requires participants to identify among the options the one representing a simple shape which is contained within the target stimulus. There are 12 items of increasing complexity (score range 0–12).

##### Mental rotation

In the mental rotation task^43,44^, participants are presented with a target stimulus shaped as the capital letter L or S, with small white or black circles at the extremities. A six-choice display includes the image corresponding to the target stimulus, rotated on the horizontal plane by 45°, 90°, 135°, or 180°, and five distractors at different degrees of rotation. The task requires participants to indicate the option matching the target stimulus after a mental rotation on the horizontal plane. There are 9 items of increasing complexity (score range 0–9).

### Statistical analysis

First, we tested differences between women and men on the two visuospatial tasks and on AQ subscales by performing a MANOVA with sex as a between-subject factor.

To assess the degree of association between social and non-social traits and the two visuospatial abilities, Pearson correlation coefficients were computed between variables.

To investigate if figure disembedding and mental rotation abilities dissociate with respect to social and non-social autistic traits and if the effect is moderated by the sex, two hierarchical multiple regressions were carried out.

In each regression, figure disembedding or mental rotation was regressed on age (as control variable), sex and the two types of autistic traits. In both regression models, all variables were included as z-scores, while sex was dummy coded (men = 0; women = 1). In the first step, age was included. In the second step, sex, AQ-social and AQ-attention were added. In the third step, the two-way interaction effects were added. In the fourth and final step, the three-way interaction was included.

When significant, the interaction effects were investigated by applying simple slope analysis and the Johnson and Neyman’s (JN) approach^50^ to define the lower and upper values of the moderator for which the effect of the predictor on the dependent variable was significant. All the analyses were performed with the psych^51^ and the interaction packages implemented into R 3.6.1 software^52,53^.

## Results

Accuracy on the visuospatial tasks and scores on AQ, separately for women and men, as well as significant differences between the two groups, are reported in Table 1.

**Table 1:** Scores (mean and SD) on visuospatial tasks and AQ, separately for women and men.

	Women		Men	
	Mean	SD	Mean	SD
**Visuospatial tasks**				
Figure disembedding	9.9	2.1	10.1	2.2
Mental rotation	6.4	2.3	6.7	2.1
**AQ**				
Total	17.6	5.7	18.1	5.6
Social skill (AQ-social)	2.2	2.2	2.1	1.8
Attention switching**	4.4	1.9	4.8	1.8
Communication	2.4	1.9	2.5	1.7
Imagination***	2.7	1.6	3.3	1.7
Attention-to-detail (AQ-attention)*	5.9	2.4	5.5	2.5

*N* = 206 women, 220 men; *p = 0.044; **p = 0.029; ***p = 0.0001.

### Correlations

Results of the correlations analysis (Table 2) showed that figure disembedding was strongly associated with mental rotation performance. Moreover, data showed that figure disembedding was significantly associated with the social autistic traits, although the effect size was small. No other significant association (p > 0.05) between visuospatial abilities and autistic traits was observed.

**Table 2:** Correlations among social traits, non-social traits, mental rotation and figure disembedding performance.

Variables	1	2	3
1. AQ-social	–		
2. AQ-attention	0.042	–	
3. Mental rotation	0.075	0.026	–
4. Figure disembedding	0.108*	0.037	0.431***

*p < 0.05. ***p < 0.001.

### Regression models

As regards the figure disembedding task, results of the hierarchical regression analysis did not show significant additive effects across the steps (Table 3).

**Table 3:** Hierarchical multiple regression analyses predicting visuospatial abilities from age, sex, social and non-social autistic traits, and their interaction.

Predictor	Visuospatial ability					
	Mental rotation			Figure disembedding		
	*R*^*2*^_*diff*_	*b* [95% *CI*]	*β*	*R*^*2*^_*diff*_	*b* [95% *CI*]	*β*
**Step 1**	0.002			0.007		
Age		0.046 [− 0.057; 0.149]	0.042		0.084 [− 0.011; 0.179]	0.084
**Step 2**	0.013			0.014		
Age		0.036 [− 0.067; 0.139]	0.034		0.079 [− 0.016; 0.175]	0.079
Sex (F = 1)		− 0.375 [− 0.800; 0.049]	− 0.085		− 0.171 [− 0.563; 0.222]	− 0.042
AQ-social		0.167 [− 0.043; 0.378]	0.076		0.219* [0.025; 0.413]	0.107*
AQ-attention		0.069 [− 0.146; 0.286]	0.031		0.073 [− 0.126; 0.273]	0.035
**Step 3**	0.007			0.012		
Age		0.035 [− 0.068; 0.138]	0.032		0.079 [− 0.017; 0.174]	0.079
Sex		− 0.377 [− 0.802; 0.048]	− 0.086		− 0.167 [− 0.558; 0.225]	− 0.041
AQ-social		0.335* [− 0.033; 0.637]	0.152*		0.388** [0.109; 0.666]	0.190**
AQ-attention		0.021 [− 0.284; 0.326]	0.009		0.013 [− 0.268; 0.294]	0.006
Sex × AQ-social		− 0.355 [− 0.782; − 0.073]	− 0.116		− 0.277 [− 0.672; 0.117]	− 0.097
Sex × AQ-attention		0.104 [− 0.329; 0.537]	0.032		0.123 [− 0.276; 0.522]	0.041
AQ-social × AQ-attention		0.084 [− 0.141; 0.308]	0.037		− 0.147 [− 0.355; 0.060]	− 0.070
**Step 4**	0.016**			0.006		
Age		0.031 [− 0.071; 0.134]	0.029		0.077 [− 0.019; 0.172]	0.077
Sex		− 0.402 [− 0.824; 0.020]	− 0.091		− 0.182 [− 0.573; 0.210]	− 0.044
AQ-social		0.337* [0.037; 0.637]	0.153*		0.389** [0.112; 0.668]	0.191**
AQ-attention		0.025 [− 0.278; 0.328]	0.011		0.015 [− 0.265; 0.296]	0.007
Sex × AQ-social		− 0.456* [− 0.888; − 0.025]	− 0.149*		− 0.337 [− 0.736; 0.063]	− 0.118
Sex × AQ-attention		0.109 [− 0.321; 0.539]	0.034		0.126 [− 0.272; 0.525]	0.043
AQ-social × AQ-attention		− 0.222 [− 0.544; 0.099]	− 0.098		− 0.326* [− 0.624; − 0.028]	− 0.154*
Sex × AQ-social × AQ-attention		0.591** [0.144; 1.038]	0.192**		0.346 [− 0.068; 0.760]	0.121
Total R^2^	0.038*			0.039*		

*N* = 426. Age = age of participants in years (z-score); Sex = participants’ sex dummy coding (Men = 0; Women = 1); *p < 0.05; **p < 0.01; ***p < 0.001.

**Figure 2:**
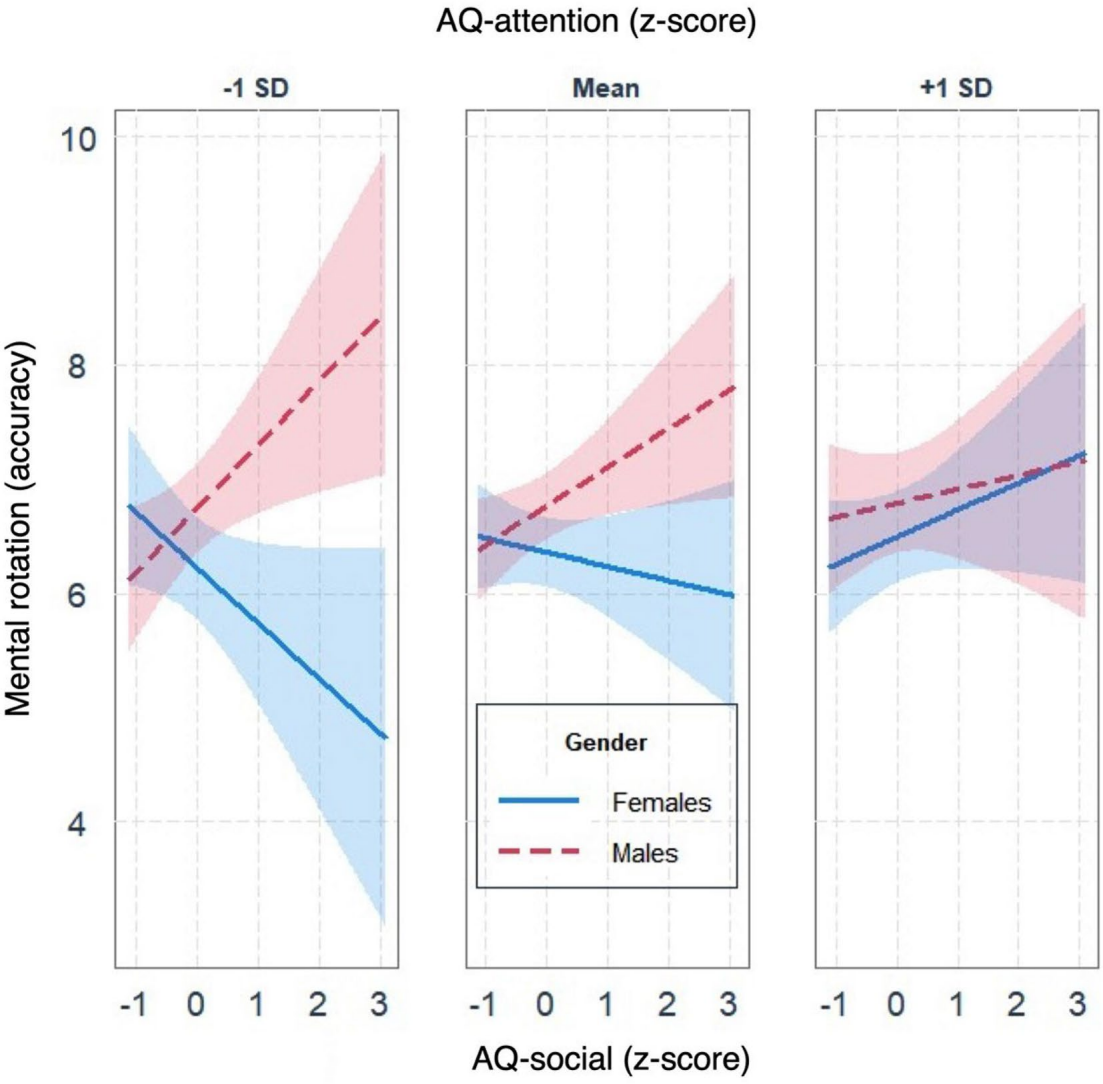
The effect of social traits on mental rotation performance moderated by both sex and non-social traits.

The regression analysis on the mental rotation task showed that the model including the three-way interaction increased the prediction of the dependent variable significantly, although the effect size was small, *R*^2^_*diff*_ = 0.016; *F*(1, 415) = 6.759, *p* = 0.009 (see Table 3). Parameter analysis of the final model revealed that, independently of the other variables in the model, the effect of AQ-social on mental rotation was moderated by both sex and AQ-attention. In particular, data showed that if non-social traits were below the mean (− 1 SD), the effect of social traits on mental rotation was positive for men (higher accuracy), whereas it was negative (lower accuracy) for women (Fig. 2). The JN analysis indicated that, when non-social autistic traits were below the mean (− 1 SD), sex differences were significant when the social autistic traits were lower than − 1.70 SD from the mean or higher than 0.09 SD from the mean. When non-social traits were on the mean, sex differences in the effect of social traits on mental rotation were similar to what was described above but attenuated. Indeed, the JN analysis indicated that sex differences were significant when social traits were higher than 0.04 SD from the mean. Instead, when non-social traits were above the mean (+ 1 SD), no sex differences were observed as regards the effect of the social traits on the mental rotation. In other words, the interaction between social and non-social traits regulated sex differences in mental rotation performance.

## Discussion

The present study found that social traits differently impact sex differences in mental rotation in people with high, middle, or low non-social traits. More precisely, when non-social traits were above the mean (+ 1 SD), no sex differences in mental rotation were found. In contrast, below this value, sex differences depended on the levels of social traits, with men of middle-to-high social traits on average outperforming women, and with women on average outperforming men with lower social traits.

On figure disembedding task, there was no significant interaction between sex and traits, but only a small positive correlation between disembedding accuracy and AQ-social score. This last finding is consistent with Russell-Smith et al.’s^11^ data showing that such a relationship, albeit weak, seems independent from sex. However, DiCriscio and Troiani^37^ found that the positive relationship between figure disembedding ability and social autistic traits was specific for men. Thus, although the present results would support the positive relation between figure disembedding ability and social autistic traits, they do not allow to clarify the role of sex differences and recommend further investigation^54^. Consistently, although the hierarchical multiple regression showed a significant AQ-social x AQ-attention interaction, it was not discussed here to avoid increasing the type I error in interpreting the results given that the delta of the R-square of the step 4 was not significant. Although this choice could correspond to a type II error, a replication of this effect is needed before interpreting it as relevant.

Regarding the involvement of different strategies in mental rotation depending on the interaction between sex and autistic traits, Stevenson and Nonack^15^ studied eye fixations during mental rotation in women and men with low, medium, and high autistic traits, as indexed by AQ total score. Their results showed that fixations by the participants in the high autistic traits group varied by sex, while no sex differences were found for medium and low autistic traits. The present data indicate that the extent to which people differ in the degree of social and non-social autistic traits is important in understanding sex differences in mental rotation.

Some authors propose that mental rotation can be accomplished by a global (holistic) or a local (piecemeal) strategy^55,56^. The holistic strategy seems related to better performance, whereas the piecemeal one to poorer performance^57^. Men on average may use a holistic strategy, whereas women on average may use a piecemeal strategy, although such differences have not been systematically confirmed^58,59^. Others have suggested that rather being divided into global vs. local, mental rotation strategies may be best divided based on their efficiency^60–62^. Across several studies, Just and Carpenter^61^ demonstrated that mental rotation can be conceived as a multi-step process in which the result of each step is monitored to determine if the intermediate result is approaching the final output. The authors also suggested that individuals performing better (high spatial individuals) are those who can keep track of the process, while individuals performing worse (low spatial individuals) are less able to keep track of the intermediate products of their partial rotations, so that their accuracy drops, especially at increasing task complexity.

Just and Carpenter’s^61^ perspective nicely fits the concept of systemizing according to which individuals with high systemizing traits are able to understand a system by identifying the key variables of the system, then manipulating them systematically and checking the effect of such manipulation on the system through *‘if-and-then’* rules^41,42^. Thus, solving a problem through systemizing implies, as a first step of the process, detecting the elements of a complex whole (the system), then exploiting them in terms of *‘if-and-then’* rules, a multi-step process allowing system-understanding and problem solving^41,42,63^. One could speculate that people with higher systemizing or high non-social autistic traits are more able to apply this multi-step process, thus showing a good performance. In contrast, people with low systemizing or low non-social traits may be less able to implement this process, leading to poorer performance. However, importantly, our results showed that when non-social traits were below the mean there was the strongest sex difference in mental rotation, with women on average being significantly less accurate than men at higher levels of social traits. Therefore, in men the effect of both non-social and social traits on mental rotation performance was positive, while in women the effect of non-social traits was positive and that of social traits was negative.

Dissociable effects of social and non-social traits on cognitive performance in women and men has been previously reported^37,48^. For instance, Davis et al.^48^ found an association between higher social traits and poorer face recognition on average in women but not in men, and between higher social traits and less looking at eyes during a face learning task in men but not in women. This is consistent with our results in that both suggest that higher social traits can be related to better or poorer cognitive performance depending on the participant’s sex. On this basis, we could conjecture that in men both social and non-social autistic traits favour the activation of an effective multi-step mental rotation strategy, whereas in women an opposite effect of the two types of traits is produced on this strategy. While the mechanism behind this sex difference warrants investigation, the merit of the present study lies in that it revealed a complex interaction between social and non-social traits, sex and mental rotation performance.

There are some limitations that require comment. First, we used visuospatial tasks that, although well-validated, did not provide a reliable and valid measure of the participants’ response times. In particular, the present paper-and-pencil mental rotation task did not allow us to directly test specific mental rotation strategies as in studies employing the computerized three-dimensional mental rotation task^64^. If the effective multi-step mental rotation strategy is effortless in individuals with high non-social traits, whereas it is effortful and time consuming in individuals with low non-social traits, one might expect to see the multi-step strategy associated with longer reaction times in people with low compared to high non-social traits. Future studies could test this prediction by using an analogous research protocol as the present one but replacing our paper-and-pencil mental rotation test with computerized versions of the task^65^.

Second, we measured non-social autistic traits with the AQ attention-to-detail subscale and assessed mental rotation that is a proxy to systemizing^17,41^. Strong systemizing has been conceived a way of explaining all the non-social features of autism, such as narrow interests, repetitive behaviour, resistance to change, and sensory hypersensitivity^63^. Thus, the relationship we found here between AQ-attention and mental rotation could be extended to other non-social autistic traits. However, this hypothesis needs to be directly verified by specific investigation.

Finally, we used Baron-Cohen et al.’s^7^ model of AQ to measure social and non-social autistic traits since its validity has been verified for the Italian version of the scale^45^. However, we must acknowledge that English et al.’s^46^ results indicated that other AQ models, such as the Russell-Smith et al.’s^36^ model, may provide more psychometrically sound factor structure of the AQ. In the light of this evidence, the present results should be replicated using other AQ models.

In summary, the present findings support the view that people with high non-social autistic traits show hyper-systemizing, consistent with the talent in autistic people when dealing with systemizing domains^41,63^. In addition, since neurotypical women, on average, show lower systemizing than men^40^, this might explain why men, on average, tend to outperform women on visuospatial tasks, such as mental rotation^12,20^. However, although men are generally higher than women in systemizing, not all men are high-systemizers and not all women are low-systemizers^13,21,41^, and the present findings indicate that when non-social traits are comparably high in women and men, a similar mental rotation performance can be observed.

In conclusion, this study sheds light on the conflicting pattern of results in literature on the influence of autistic traits on mental rotation, paving the way for future investigations of this.
